# Implications of COVID-19 Pandemic on the Emergence of Antimicrobial Resistance: Adjusting the Response to Future Outbreaks

**DOI:** 10.3390/life11030220

**Published:** 2021-03-10

**Authors:** Doris Rusic, Marino Vilovic, Josipa Bukic, Dario Leskur, Ana Seselja Perisin, Marko Kumric, Dinko Martinovic, Ana Petric, Darko Modun, Josko Bozic

**Affiliations:** 1 Department of Pharmacy, University of Split School of Medicine, Soltanska 2, 21 000 Split, Croatia; drusic@mefst.hr (D.R.); jbukic@mefst.hr (J.B.); dleskur@mefst.hr (D.L.); aperisin@mefst.hr (A.S.P.); ana.petric@ljekarnasdz.hr (A.P.); dmodun@mefst.hr (D.M.); 2 Department of Pathophysiology, University of Split School of Medicine, Soltanska 2, 21 000 Split, Croatia; mvilovic@mefst.hr (M.V.); marko.kumric@mefst.hr (M.K.); dinko.martinovic@mefst.hr (D.M.); 3 Split-Dalmatia County Pharmacy, Kneza Ljudevita Posavskog 12 b, 21 000 Split, Croatia

**Keywords:** COVID-19, coronavirus, antimicrobial resistance, ICU, biocide, disinfection, antibiotics

## Abstract

The net effect of the coronavirus disease 2019 (COVID-19) pandemic and the response to it on the emergence of antimicrobial resistance is yet unknown. Positive impacts on the spread of multiresistant pathogens and infections in general may be observed with the implementation of general preventative measures for the spread of infectious disease such as social distancing, reduced travel and increased personal hygiene. This pandemic has accelerated the development of novel technologies, such as mRNA vaccines, that may be used to fight other diseases. These should be capitalized upon to manage the ongoing antimicrobial resistance pandemic in the background. However, it is likely that the COVID-19 pandemic is fueling the emergence of antimicrobial resistance due to high rates of inappropriate antimicrobial prescribing, the high use of biocides and the interruption of treatment for other conditions. Clinical uncertainty driven by the lack of effective diagnostics and practice of telemedicine may have driven the inappropriate use of antimicrobials. As pathogens know no borders, increased focus is needed for infectious diseases still threatening low- and middle-income countries such as tuberculosis. Stewardship measures for future outbreaks should stress the importance of social distancing and hand washing but discourage the overuse of disinfectants and antimicrobials that are not proven effective.

## 1. Introduction

The World Health Organization declared the coronavirus disease 2019 (COVID-19) a pandemic in March 2020 [1]. In the shadows of the COVID-19 pandemic, there has been an ongoing antimicrobial resistance pandemic [2,3]. One of the modifiable elements contributing to the emergence of the antimicrobial resistance is the unnecessary and excessive use of antimicrobials [4,5,6]. It is likely that the COVID-19 pandemic is fueling the emergence of antimicrobial resistance due to high rates of inappropriate antimicrobial prescribing, a high use of biocides and the interruption of treatment for other conditions [7]. However, in contrast to the collective action problems, such as antimicrobial resistance, the COVID-19 pandemic is an immediate and ongoing crisis [8]. The World Health Organization has stressed the importance of integrating antimicrobial stewardship measures into the COVID-19 response of the healthcare system. The key competencies needed are the diagnosis of severe COVID-19 with coinfections, avoidance of the unnecessary use of antimicrobials, utilization of medical devices that reduce the spread of hospital-acquired infections and adopting infection prevention and control measures [7]. Moreover, the World Health Organization does not support the use of antimicrobials for mild cases of COVID-19 with a low suspicion of bacterial coinfection. It does however state the use of empiric antimicrobials for the treatment of all likely pathogens for suspected and confirmed severe COVID-19 cases [9]. Despite low evidence to support such practice, azithromycin, at one point, became a common treatment for COVID-19 [10].

The COVID-19 pandemic has caused a disruption in healthcare and emergency services resulting in a decreased number of patient screening exams and admissions, healthcare personnel and drug shortages and a surge in misinformation. Moreover, economic effects such as unemployment have led to the loss of insurance coverage, and the financial crisis combined with greater health expenditure has pushed many healthcare systems to their limits [11,12,13]. The debts of this disruption will likely be observed in the post-pandemic era as an increase in deaths of otherwise preventable illnesses. The disruption of healthcare for other infectious diseases and vaccinations may additionally increase the rise of infections and drive the overuse of antimicrobials [7]. The increasing consumption of antimicrobials, as well as their environmental disposal, has been pressuring the selection of multidrug-resistant strains that emerge at a fast pace, outcompeting the development of new antimicrobials [14]. The inappropriate use of antimicrobials during the COVID-19 pandemic may aggravate antimicrobial resistance to the point of more deaths as an unforeseen consequence of the COVID-19 pandemic [15].

Although the early data suggested low rates of coinfection with COVID-19, there is an indiscriminate use of antimicrobials present indicating that antimicrobial stewardship may be another casualty of the COVID-19 pandemic [16,17]. The utilization of established antibiotic stewardship programs has plummeted as healthcare workers struggle to save lives of patients with COVID-19 and are even asked to prioritize COVID-19 response and management [16,18]. According to a study conducted among hospital physicians in Greece, 98.5% stated that antimicrobial stewardship programs must be further developed during the COVID-19 pandemic [19]. In the UK, the majority of healthcare professionals believe that the COVID-19 pandemic had a negative impact on regular antimicrobial stewardship activities, and only 7% thought it had a positive impact, while 25% stated they observed both positive and negative impacts. Positive outcomes were observed as the introduction of novel biomarkers for differencing bacterial and viral infections and the greater utilization of technologies that facilitate stewardship, as well as greater collaborations [20].

This pandemic has outlined the ease at which pathogens cross borders and illustrated that the top healthcare systems are only as alert and prepared as the most vulnerable systems worldwide [15,21]. The speed of the spread of the severe acute respiratory syndrome coronavirus 2 (SARS-CoV-2) in unprepared systems for such a crisis and the absence of adequate diagnostic are mistakes that should not be repeated for the handling of antimicrobial resistance or future outbreaks of infectious diseases [22].

One of the major issues for research in infectious diseases is a lack of public interest and funding, both private and corporate, in different fields. With time, it will become evident whether the increased funding for the COVID-19 pandemic may expand to other infectious diseases. This can also be observed as a possible positive impact of the pandemic [23]. Stakeholders are being given a unique opportunity to promote and stress the likely consequences that infectious diseases may have on human health [24]. The economic devastations, alongside the weakening of healthcare systems brought on by the COVID-19 pandemic, has put infectious diseases and the importance of investment in the public health at the center of the public’s attention. This poses a unique opportunity to reconsider antimicrobial stewardship and antimicrobial development [25].

The COVID-19 pandemic and the response to the pandemic may have both positive and negative impacts on the known drivers of antimicrobial resistance. An increase in antimicrobial resistance during the COVID-19 pandemic is expected due to overcrowding of the healthcare system, an increase in empiric antimicrobial use and protective early antibiotics treatments for patients infected with SARS-CoV-2, less surveillance cultures and the diagnosis of antimicrobial resistant organisms, as well as overall weakening stewardship measures. Further, a minor impact on antimicrobial resistance is expected from the increase of adopted infection control measures for healthcare personnel, use of personal protective equipment and more frequent and detailed hand hygiene, as well as devices for the decontamination of air and surfaces [26]. Factors related to the COVID-19 pandemic that may influence its antimicrobial resistance are summarized in Table 1 [27,28,29,30,31].

The aim of this review is to discuss current knowledge and opinions on how the specific elements of the COVID-19 pandemic and the response to the pandemic may impact the emergence of antimicrobial resistance.

## 2. Antibiotic Prescribing for COVID-19 and The Unnecessary Use of Antimicrobials

The COVID-19 pandemic has taken the world by surprise, and as new knowledge arises, so does the recommendations for treatment changes. During the COVID-19 pandemic, it is reasonable to expect a higher than usual level of administration of antimicrobials, as there have been trial and error in treatments based on previous experiences with similar pathogens or potential mechanisms of action of known drugs. Antibiotic treatments are mostly applied as prophylaxis to prevent further bacterial coinfections among hospitalized patients, but, in several countries, the common and extensive use of antimicrobials in the treatment of hospitalized COVID-19 patients is considered to be a part of the routine treatment package. Furthermore, it is possible that the amounts and types of antimicrobials used are currently under-documented and, thus, underestimated [32,33]. Additionally, the hype around premature reports of possible treatments for COVID-19 may also spike the prescription of antibiotics. In example, the hype around azithromycin/hydroxychloroquine resulted in sporadic drug shortages [34]. Media reports and political leaders amplified the likely effect that azithromycin in combination with hydroxychloroquine may treat COVID-19, possibly contributing to the shortages of both drugs [35]. According to the early data from New York hospitals on 7914 patients with COVID-19, only 15.4% were not prescribed with azithromycin or hydroxychloroquine [36].

Although unmeasured, there is a chance that a proportion of people are taking antibiotics in misguided attempts to prevent infection and demanding them from physicians. Such practices would likely be more prevalent in communities where antibiotics are easily obtained without a prescription, such as developing countries [34,35]. In March 2020 in Spain, with the surge of COVID-19 cases, the consumption of antibiotics increased 11.5% relative to February of 2020. Moreover, the consumption in March surpassed the peak levels of consumption in January 2020. The consumption of azithromycin in March 2020 was at 400% from February 2020. Moreover, alongside the great consumption of azithromycin, another seven antibiotic consumptions peaked in March 2020 [37].

It appears that the prevalence of a bacterial coinfection depends on the studied country and on the time of sampling relative to the onset of symptoms. As in other infections, the first choice of antibiotics in COVID-19 patients is most often empiric. The initial response to the pandemic with antibiotic prescribing was influenced by early reports of a great prevalence of bacterial coinfections in patients with COVID-19 but, also, on previous experiences with, i.e., an influenza pandemic. However, the reported rates of bacterial/fungal coinfections are lower in COVID-19 than in other coronavirus studies [38]. The growing evidence is in favor of specific antimicrobial stewardship measures for COVID-19 rather than turning to existing empiric treatment guidelines for influenza patients with bacterial coinfections [39].

As mentioned previously, the bacterial coinfection rate in patients with COVID-19 was lower in comparison to those observed in influenza pandemics. However, reports of the use of broad-spectrum antimicrobials in COVID-19 patients are high and frequent [40]. When diagnostic options are limited and data to support clinician decision-making is scarce, the focus should be directed to infection prevention and control measures [24]. A study on 554 confirmed and 23 suspected COVID-19 patients revealed that 6% of patients got antibiotics, of which 61% were appropriate. An analysis showed that such practice resulted in 59 days of inappropriate antibiotic use. Moreover, suspected cases were started on antibiotics more frequently compared to confirmed cases of COVID-19 [41].

A study on patients with COVID-19 hospitalized in New York identified the five most common organisms in patients with COVID-19 to be *Staphylococcus aureus* in 44%, *Pseudomonas aeruginosa* in 16%, *Klebsiella* spp. in 10%, *Enterobacter* spp. in 8% and *Escherichia coli* in 4% of cases, while multidrug-resistant Gram-negative isolates were present in 15% of patients. As many as 79% of patients included in the study had antibiotic exposure prior to testing, rising to 98% that had antibiotic exposure at some time point during their COVID-19 hospitalization. An analysis showed that all patients with identified multidrug-resistant organisms had antibiotic exposure, compared to 65% of patients without multidrug-resistant organisms. The susceptibility data for different organisms were investigated and compared for the periods of March and April of 2019 and 2020. *Klebsiella pneumoniae* had a susceptibility decline of over 10% to cephalosporins, ciprofloxacin and meropenem between 2019 and 2020. There were 42% more *Enterobacteriaceae* isolates resistant to carbapenems in 2020 (12 vs. 17 isolates), likely driven by selective antibiotic pressure [42]. It is unclear in the mentioned study whether carbapenems were used prior to identifying coinfections for the treatment of COVID-19 or as a prophylaxis driven by fear that patients with SARS-CoV-2 may be more susceptible for other infections. The literature does not describe the use of carbapenems for COVID-19 per se, but it is likely that there have been such attempts in practice, especially at the very beginning of the pandemic. Interestingly, there are reports of the increase of Carbapenem-resistant *Acinetobacter baumannii* isolates in an acute care hospital in New Jersey during the early pandemic, likely due to decreased vigilance for the control of multidrug-resistant organism transmissions; reduced personnel numbers and both intentional and unintentional changes in infection prevention and control practice driven by shifting priorities, personnel and protective equipment shortages [43].

Other research describes the frequent unnecessary use of antimicrobials and mismatch with infections. In a research conducted among patients with SARS-CoV-2, 72% of patients were prescribed with antimicrobial treatments for lower respiratory tract infections. However, respiratory pathogens were identified in only 6% of patients. The practice was that antimicrobial treatment was continued after positive SARS-CoV-2 PCR testing and the absence of evidence of bacterial infections in the majority of patients. Overall, the median duration of treatments with antimicrobials in the mentioned research was seven days [44].

In medical center in the Bronx, NY during the surge of the COVID-19 pandemic, five cases of New Delhi Metallo-beta-lactamase producing *Enterobacterales* infections were diagnosed in patients initially admitted with confirmed COVID-19 pneumonia. The antibiotic use in the mentioned hospital was extensive, as 71% of COVID-19 patients admitted between 1st March and 31st May, 2020 received antibiotics, but less than 5% acquired bacterial coinfections [45]. A study from Italy revealed seven out of 35 patients had a positive rectal swab for carbapenemase-producing *K. pneumoniae*, as Italy had an increase in the spread of this pathogen prior to the COVID-19 pandemic [46].

A review of the COVID-19 studies reported 8% with bacterial/fungal coinfections. However, the high rates of antimicrobial prescribing were present (72%), mostly broad-spectrum and empiric across both critical and noncritical care settings [38]. The overuse of antimicrobials may trigger a post-COVID pandemic of multidrug-resistant strains, putting healthcare systems at additional risk [47].

A meta-analysis on 30,623 patients estimated the prevalence of antibiotic prescribing in patients with COVID-19 at 74.6% (95% confidence interval (CI) 68.3–80.0%). Fluoroquinolones were prescribed in most cases (20.0%), followed by macrolides (18.9%), β-lactam with β-lactam inhibitors (15.0%) and cephalosporins (15.0%). The prevalence of concomitant bacterial infections with COVID-19 was 8.6%. Antibiotic stewardship was reported only in 1.9% of the included studies. Antibiotic prescribing was highly prevalent in intensive care units (ICU) at 86.4%, followed with inpatient hospital settings (74.8%) and lowest in mixed inpatient/outpatient settings (59.3%). The prevalence of antibiotic prescribing rose with patients requiring mechanical ventilation. As the pandemic progressed, a trend towards reduced antibiotic prescribing was observed [48].

The debate on whether prescribed antibiotics were justified is uncalled for. However, there is a need for a strategy for the future use and off-label use of existing antibiotics in unexpected emergency situations such as the COVID-19 pandemic. Overall, antimicrobial treatments should be reserved for those with suspected or proven bacterial coinfections and frequent re-evaluations (based on clinical course laboratory findings and imaging findings) should be encouraged [40].

Although the use of broad-spectrum antimicrobials and early treatments with antibiotics may promote resistant organisms, the overall net effect may be adjusted with the lower access of healthcare to other patients and overall disruption of regular healthcare, as this is likely to reduce the overall use of antimicrobials [49]. Reports on the volumes of antibiotic use at VA Pittsburgh show that there was a 6.5% monthly reduction (CI 3.0–10.1%, *p* = 0.001) in days of therapy during March to June 2020 compared to January 2018 through February 2020. However, the overall antibiotic days of therapy per 1000 bed days of care was significantly increased. In particular, there were significant increases observed for non-antipseudomonal penicillin and macrolide (i.e., azithromycin), but azithromycin was used only as an empirical therapy for community-acquired pneumonia in patients with COVID-19, not to treat COVID-19 [50].

Inadequate testing for SARS-CoV-2 can increase clinical uncertainty [35]. The unnecessary use of antimicrobials is supported with the lack of rapid diagnostics and decision support tools for the clinical management of COVID-19. Treatment research for COVID-19 should include a search for treatments for both COVID-19 and known coinfections, as these greatly contribute to a bad prognosis. The pandemic provides a unique opportunity to capitalize on this experience and promote and further develop existing infection control, as well as raise awareness of the importance of effective antimicrobials and the role of vaccines. A potential long-term benefit could be a reduction in the use and consumption of antibiotics [49,51]. However, the experience of telemedicine in pediatric care (conducted prior to the COVID-19 pandemic) adds to the possible detrimental effects of the pandemic on antimicrobial resistance, as efforts to reduce patient–provider contact may result in the inappropriate use of antimicrobials. The lack of cost-effective, rapid diagnostics poses an additional challenge for prescribers for determining the etiology of an illness [52,53,54]. A retrospective cohort on data in 2015 to 2016 showed that, in telemedicine for the pediatric population, there has been an increase in the overprescribing of antimicrobials compared to face-to-face visits. This experience stresses the importance of stewardship strategies such as a prescription review [53]. The results from other research are inconclusive. Prescribing according to the guidelines was sustained when switching from face-to-face to remote prescribing for sinusitis, but mixed data was found for acute respiratory infections [55]. Putting these results in the perspective of the COVID-19 pandemic with the implementation of preventive measures and accounting for the decrease in community-acquired infections, it is likely that the overall antibiotics prescribed in primary and outpatient settings has decreased, but due to remote prescribing, the proportion of appropriate prescriptions may have also decreased. Experiences from dental practices in England during the pandemic showed an increase in antibiotic prescribing during restricted access to dentistry. The prescribing of antibiotics was 25% higher from April to July of 2020, compared to the same months of 2019 [56]. One possible reason may be that several urgent dental centers required patients to opt for antibiotics prior to face-to-face visits [57].

The unnecessary use of antimicrobials in patients with COVID-19 may be further promoted with illness presentation, as antibiotics are frequently administered for acute, uncomplicated diarrhea, which promotes antimicrobial resistance with marginal benefits to the diarrhea in general. The further unnecessary use of antimicrobials arose from the early treatments for COVID-19, such as the use of chloroquine (or hydroxychloroquine) with azithromycin. With the assumption that 50% of patients with COVID-19 required hospitalization and 10–70% of those needed critical care that included antimicrobial treatment, there was an estimate of up to 10.5 million antimicrobial treatments administered for COVID-19 alone, most in combinations and during prolonged hospital stays. Furthermore, estimating that outpatient prescribing for acute respiratory infections during the COVID-19 pandemic will follow the observed trends of prescribing antibiotics, although 90% cases are of a viral etiology, this would result in an additional 10.8 million antibiotic treatments for patients with COVID-19 or even more, taking into consideration that the pandemic is still ongoing [58].

Another undesirable effect of the use of antibiotics in patients may be alterations in the gut microbiota. Drugs that were sometimes used for prophylaxis or the treatment of COVID-19 are known to promote an increase of the proportion of resistant *Enterococci* in the gut microbiota. As far as azithromycin is concerned, there are reports of a reduction in the diversity of microbiota in patients with asthma, as well as increases in resistance gene carriage in viridians streptococci [59,60]. Moreover, disturbances in the gut microbiota may facilitate worse outcomes in patients with COVID-19. Interestingly, there are reports that COVID-19 patients with gastrointestinal (GI) complications experience greater respiratory distress compared to COVID-19 patients without GI symptoms [61,62]. Furthermore, the link between the host’s immune system and gut microbiota is well-established, and studies show decreased levels of probiotic bacteria in COVID-19 patients. This dysbiosis is further aggravated with other pathogens, such as bacterial coinfections, leading to a surge in proinflammatory cytokines [62].

However, some positive experiences came from a hospital in Singapore with an established multidisciplinary antimicrobial stewardship team. At intensive care wards, the proportion of patients receiving antimicrobials decreased from 2015 to 2018. In 2019, the proportion of patient receiving antimicrobials spiked to a high of 81%, but in 2020, the antimicrobial use at COVID-19 wards was 29% [63].

### 2.1. Infections in the Intensive Care Units

Among the initial recommendations for the treatment of patients presenting COVID-19 symptoms in intensive care units were empirical broad-spectrum antibiotics and neuraminidase inhibitors [64]. However, the use of broad-spectrum antibiotics can promote *C. difficile* infection and a rise in antimicrobial resistance [16].

An experience from intensive care units in Maryland, USA showed a rapid spread of multidrug-resistant bacteria among patients with COVID-19 during May–June 2020. High antibiotic consumption, critical illness, overcrowding and low compliance of prevention practices were recognized as likely contributors to the spread [65]. Researchers from Italy reported a nosocomial outbreak of *Candida auris* and a high rate of colonization/infection with carbapenem-resistant *P. aeruginosa* occurring in ICUs at one of the COVID-19 dedicated hospitals. The authors suggested that the main drivers of an increased antimicrobial resistance during the COVID-19 pandemic in the hospital were the horizontal spread of resistant strains and the use of broad-spectrum antimicrobials. The wide prescription of broad-spectrum antibiotics is likely due to the difficulty in differentiating among pulmonary bacterial coinfection and viral infection alone. Further, they speculated that the severity of clinical conditions in a setting with a high rate of extended-spectrum beta-lactamases-producing bacteria might have contributed to the extensive use of carbapenems in empirical therapy. Additionally, they outlined that the contact isolation measures utilized during the COVID-19 pandemic might have been suboptimal, as protective equipment was used mainly to protect healthcare personnel from viral infection, often neglecting avoiding pathogen transmissions between patients. Shortages of such equipment may have further caused suboptimal use and infrequent renewal. Moreover, in COVID-19 ICUs, the use of open spaces was preferred to allow an easier management of a great number of patients, increasing the likelihood of horizontal transmission [66].

The experiences of ICUs during the pandemic are somewhat mixed. Reports from ICUs indicate the incidence or resistant infection acquisition at 30/1000 patients-day. The data shows that, of 72 patients, 33% acquired 31 multidrug-resistant bacteria during their stay at the ICU [67]. These high rates of acquisition of multiresistant infections may be explained with the work overload and ICU overcrowding, as well as a decreased adherence to control measures and shortages of personal protective equipment [25,27,28,51,68].

Intubation, especially for longer periods of time, such as seen in critically ill patients with COVID-19, is a known risk for hospital-acquired infections. Furthermore, hospital data shows a continuous increase in multidrug-resistant Gram-negative bacteria, which can lead to potentially deadly coinfections in patients with COVID-19 [35]. In Wuhan, China, during the first documented outbreak of the new COVID-19 disease, ventilator-associated pneumonia occurred in 31% of patients under invasive mechanical ventilation [69]. At an ICU in New Delhi, of 420 patients with COVID-19 that required mechanical ventilation, 2.5% developed candidemia [70]. In another study, of 19 COVID-19 patients admitted to an ICU, all were positive for bacterial infection, 90% *A. baumannii* and 10% for *S. aureus* strains [71].

A case-control study conducted on the data from a hospital in Rome showed an overall lower incidence of multidrug-resistant bacterial infections during the COVID-19 pandemic compared to previous years. However, COVID-19 patients have multidrug-resistant infections significantly more frequently than non-COVID-19 patients (19.2 vs. 29.3. cases per 100 discharges, respectively). The highest increase was observed for extended-spectrum β-lactamase *K. pneumoniae* (4.8 vs. 10.6 cases per 100 discharges). Obviously, the maintenance of a high level of preventive measures for infectious disease lowers the spread of multidrug-resistant bacteria [72].

The results of a study conducted among 260 patients in Egypt indicated around 10% of patients with COVID-19 had bacterial or fungal infections [73]. According to a meta-analysis on 3834 COVID-19 patients, 7% of hospitalized patients had a bacterial coinfection, with higher proportions observed at ICUs. The most common pathogens were *Mycoplasma pneumoniae*, *P. aeruginosa* and *Haemophilus influenzae* [74].

The prevalence of carbapenemase-producing *Enterobacterales* in New York City declined substantially in the recent years, but the increased detection among COVID-19 patients may signal its re-emergence [75]. Experience from a teaching hospital that has implemented antimicrobial stewardship measures at an ICU with special attention on infection control measures, the carbapenem-resistant *Enterobacteriaceae* incidence rose from 6.7% in 2019 to up to 50% in March and April of 2020. Obviously, with staff training and implementation of the antimicrobial stewardship measures, a reduction in carbapenem-resistant *Enterobacteriaceae* was expected [76].

In a hospital with active ongoing antimicrobial stewardship programs, the empirical treatment for pneumonia at hospital admission was administered to 33.7% of patients with COVID-19. The antimicrobial consumption increased at a rate of +3.5% for six weeks after the national lockdown, followed by a weekly reduction of −6.4%. There was no significant change in the incidence of hospital-acquired candidemia or multidrug-resistant organisms associated with hospital-acquired bloodstream infections [77].

Viral infections may make patients more susceptible to secondary bacterial infections that can become more invasive and life-threatening than the initial illness. The misuse of antimicrobials in COVID-19 patients, i.e., azithromycin may trigger bacterial coinfections with resistant strains [37].

### 2.2. Other Infections

The changing epidemiological profile of infectious diseases due to the response to the COVID-19 pandemic resulting in the reduced transmission of other respiratory tract illnesses has likely influenced the decline in antibiotic prescribing observed in general practitioners’ services. This may be a result of changed patient health-seeking behaviors more than changed clinician-prescribing behaviors. Unfortunately, the proportion of broad-spectrum antibiotics in the total prescribed antibiotics has increased [78].

During the COVID-19 pandemic, efforts made against other deadly infectious diseases may have diminished. For example, there are expected to be 6.3 million new cases and 1.4 million deaths caused by tuberculosis each year until 2025, setting the efforts to fight the world’s leading infectious disease killer further back [79]. Moreover, according to research in Korea, the treatment success rate decreased from 90.5% before COVID-19 to 84.6% after COVID-19, with a percentage change of −6.5% at the national level [80].

As patients with COVID-19 disease may present with various symptoms mimicking other common acute respiratory conditions, diagnostics are crucial for treatment optimization in the emergency department but are also limited and lacking. Experience with other respiratory conditions with less diagnostic uncertainty than COVID-19 has shown a high prevalence of antibiotic misuse despite established guidelines that discourage the use of antibiotics in conditions such as influenza and bronchitis. Taking into consideration the challenges of diagnosing COVID-19, especially during the first wave, it can be expected that there was a substantial overuse of antimicrobials and unnecessary antibiotics prescribed during admission, increasing the pressure for the selection of drug-resistant infections. Another factor likely supporting high antibiotic prescribing rates for patients with COVID-19 is the large proportion of patients with early hypoxia and progression to respiratory failure [81].

The possible stewardship measures in emergency departments may include the use of rapid, viral pathogen-detection assays to support the discontinuation of antibiotic treatments when appropriate, the development of care pathways/guidelines and the review of empirical antibiotic selections. Furthermore, this pandemic has stressed the urgent need for identifying the differences of host response biomarkers relative to bacterial or viral infections in patients presenting with symptoms of acute respiratory illness [81]. Research indicates that procalcitonin may be a useful guide for the de-escalation of antibiotics and a useful antimicrobial stewardship tool, as the utilization of procalcitonin levels in COVID-19 patients to indicate possible bacterial infections significantly reduced antibiotic use by two days [82].

A coinfection with antimicrobial-resistant pathogens have been reported in patients with COVID-19. Namely, infections including *K. pneumonie*, *P. aeruginosa*, extended-spectrum beta-lactamase, multidrug-resistant *E. coli*, *Enterococcus*, *Chlamydia pneumoniae*, *M. pneumoniae* and *Acinetobacter* [83].

According to the research by Fattorini et al., the most common isolated bacteria in patients with COVID-19 were *M. pneumoniae*, *S. aureus*, *Legionella pneumophila*, *Haemophilus* spp., *Klebsiella* spp., *P. aeruginosa*, *Chlamydia* spp., *S. pneumoniae* and *A. baumannii*. Among patients in the ICU, 1.3% of them developed superinfections with resistant strains of *S. aureus*, *K. pneumoniae, P. aeruginosa* or *A. baumannii*. Frequent tuberculosis–COVID-19 coinfections were also reported. A literature analysis by Fattorini et al. showed that 88.3% of COVID-positive patients received treatment with broad-spectrum antibiotics [84].

In a study on 102 patients with COVID-19 and secondary bacterial infections, 159 strains of bacteria were isolated. The top three were *A. baumannii*, *K. pneumoniae* and *Stenotrophomonas maltophilia*. The mixed infections were most frequently *A. baumannii* with *K. pneumoniae*. There were 91.7% carbapenem-resistant *A. baumannii* and 76.6% carbapenem-resistant *K. pneumoniae*, 100% methicillin-resistant *S. aureus* and 75% extended-spectrum beta-lactamase-producing *E. coli* [85].

## 3. Personal Protective Equipment and Hygiene Practices

In the time period between the 26th of February 2020 and 20th of March 2020, a shortage of personal protective equipment supplies such as face masks was reported by 48% of healthcare professionals (ranging from 64.2% in low-income countries to 27.4% in high-income countries) [86]. Moreover, the ongoing outbreak resulted in shortages of rubbing alcohol and commercial alcohol-based sanitizers as well [87].

Adequate hand hygiene is the first line of defense against the COVID-19 pandemic. In 20 s, the surfactants in soaps dissolve the lipid bilayer, an integral part of the SARS-CoV-2 virus envelope, thus deactivating the virus. These viral molecules are encapsulated within micelles and washed away. All soaps available in the marketplace have the basic ingredients required to deactivate the virus; therefore, antibacterial soaps are unnecessary. However, there was a sharp increase in the sale of antibacterial soaps and disinfectants worldwide, indicating their increased and unnecessary use [34]. Hygiene practices, such as those implemented during the COVID-19 pandemic, may slow the dissemination of antimicrobial resistance, but conversely, they can support emerging drug resistance resulting from the elevated consumption of such antimicrobial products, i.e., biocides. The greater use of these products leads to higher concentrations of biocide-based products in soil, water, ponds and, more importantly, in the microecosystems and microecological niches where various bacterial species are present, underground waters and wastewater treatment systems. It is presumed that high concentrations of such microbicidal agents could kill most bacterial species that provide beneficial services to the ecosystem and to other living organisms. Furthermore, if the concentrations of microbicidal agents are at the subminimum inhibitory concentration, this may augment the selective pressure and drive the emergence of antimicrobial resistance [33].

The overuse of alcohol-based hand sanitizer promotes antimicrobial resistance, as well as the use of nonantibiotic agents, such as biocides or toxic substances, that can also contribute to this pressure by favoring the selection/development of a resistance to specific antibiotics [88,89]. The extent of the use of sanitizers and disinfectants during the COVID-19 pandemic is immeasurable. Therefore, the exact effects of such immersive use on the microbiomes of humans, animals and the environment are yet unknown and immeasurable. Dysbiosis in host–commensal interactions may be an outcome of such practices, affecting not only the host’s immune functioning but, also, the susceptibility to infectious and noninfectious diseases. Probiotics and similar preparations are possible correctives for dysbiosis, but the disruption caused by the excessive use of sanitizers and disinfectants worldwide extend beyond dysbiosis. For example, there has been a recently reported emergence of alcohol resistance, a 10-fold increase in resistance to handwashing alcohol (70% isopropanol), in *E. faecium*, the leading cause of nosocomial infections [90,91]. Furthermore, there are reports of *E. coli* (48%) and *P. aeruginosa* (64%) isolates resistant against all available sanitizers on the market [92]. Other reports also indicate that the excessive use of alcohol-based hand sanitizers in inpatient settings promotes resistant strains of pathogens [93,94]. Several pathogens, including *E. coli*, *P. aeruginosa*, *B. cereus* and *Mycobacterium* species, have shown tolerance and resistance to benzalkonium chloride. A cross-resistance between benzalkonium chloride and antibiotics has also been documented [94,95]. Moreover, the excessive use of surfactants, alcohol and hydrogen peroxides are also known to cause resistance in microorganisms [91,94,96,97].

The current wastewater treatment techniques are not effective enough to offer the complete elimination of antibacterial biocides [34]. The uncontrolled use of biocide-based products during the COVID-19 pandemic will potentially lead to an unusual and increased release of such antimicrobials into the environment, which will pressure the selective survival of resistant bacterial strains [98,99].

An emphasis on hygiene measures, stressing more frequent handwashing, seems to obtain results and is especially highlighted in the COVID-19 pandemic. This adopted behavioral change, in the long run, may have a positive impact on the emergence of antimicrobial resistance [7,100]. Additionally, a massive-scale reduction of international travel is expected to have also slowed the spread of multiresistant organisms [51]. A targeted hygiene approach in people’s everyday lives is one way to maximize the protection against infection during the times and situations where there is the greatest risk of transmission of the pathogen. This, in turn, leads to a reduced need for antibiotics, thereby minimizing the selection pressure for resistant pathogens. Many community-onset infections are often associated with recent discharges from an inpatient setting. Multidrug-resistant strains can spread to other family members, who are then infected or colonized, which does not necessarily result with a clinical presentation of the disease. Experience with the spread of methicillin-resistant *S. aureus* (MRSA) from a colonized individual at a home to other family members showed that antibiotic treatments may fail in eradication due to the recolonization from contaminated environmental surfaces. Eradication is successful only when antibiotic treatment is combined with rigorous cleaning of the environment. Laboratory studies have linked microbicide use with the reduction in antibiotic susceptibility. However, these observations may not translate to real-life clinical practice, and further research is needed [101]. Moreover, the COVID-19 pandemic may serve as a nature experiment. It is important to outline that preventing viral infections, as well as bacterial infections—especially those that may cause respiratory and gastrointestinal infections—can also play a role in reducing the antimicrobial resistance, as this will eliminate the occurrence of illness and, thus, the potential for the mis-prescribing and misuse of antibiotics [101].

## 4. Impact on Scientific Research

Vaccines are an effective tool to reduce the demand for antimicrobials, thus slowing the spread of resistant pathogens. The research shift caused by the COVID-19 pandemic resulted in some great benefits for future public health, as it has accelerated the maturation of viral and RNA vectors by at least a decade. The urgent need for new vaccines has accelerated the speed of developing vaccines and the availability of new powerful technologies, making new platforms available for other health priorities, such as cancer and other infections [102]. A positive impact can be seen on personal protective equipment development as well. A new face mask filter treated by dip coating with 70% ethyl alcohol containing 0.1% benzalkonium chloride showed high antibacterial activity against MRSA (methicillin-resistant *S. aureus*) and MRSE (methicillin-resistant *S. epidermidis*) and antiviral activity against SARS-CoV-2 [103].

On the downside, the emergence of the COVID-19 pandemic has affected the research of vaccines and treatment options for other diseases. New antimicrobials and vaccines research and development for other infectious diseases are on-hold, and ongoing clinical trials are suspended [12,83].

## 5. Conclusions

In recent years, the Western world has mostly focused on noncommunicable diseases. The COVID-19 pandemic has put the somewhat forgotten infectious diseases back into the spotlight of the developed world. Positive impacts on the spread of multiresistant pathogens and infections in general may be observed with the implementation of general preventative measures against the spread of infectious disease such as social distancing, reduced travel and increased personal hygiene. Moreover, the pandemic has accelerated the development of novel technologies, such as mRNA vaccines, that may be used to fight other diseases. These should be capitalized upon to manage the ongoing antimicrobial resistance pandemic in the background. However, clinical uncertainty driven by the lack of effective diagnostics and the practice of telemedicine may have driven the inappropriate use of antimicrobials. Furthermore, the increased focus and funding of research may accelerate the development of new diagnostic tools and effective treatments, such as vaccines, that may ease the pressure on ICUs and lower the spread of multiresistant organisms. Future stewardship measures for new infectious disease outbreaks should stress the importance of social distancing and hand washing but discourage the overuse of disinfectants, biocides and antimicrobials that are not proven effective (i.e., azithromycin for COVID-19). Furthermore, the research for novel treatments should be supported. The COVID-19 pandemic and antimicrobial resistance have multiple interactions, but how they will unfold is yet to be discovered. Finally, greater efforts are required to preserve antimicrobial stewardship measures during both the COVID-19 and future outbreaks.

## Figures and Tables

**Table 1:** Factors related to the coronavirus disease 2019 (COVID-19) pandemic that may influence its antimicrobial resistance.

Factors Favouring the Emergence of Antimicrobial Resistance	Factors Favouring a Decrease in Antimicrobial Resistance
Increased use of biocidal agents in the environment Halt in research for other infectious diseases Unlicensed use of some agents Self-medication fuelled by the media covers and demand of potential treatments (i.e., hydroxychloroquine) Drug (especially narrow spectrum antimicrobials) and personal protective equipment shortages Increased rate of empirical antimicrobial treatment for respiratory illness Overcrowding and overloading of healthcare systems	Increased focus on hand hygiene Social distancing Reduced travel Decreased incidence of infections due to social distancing, enhanced hygiene, disinfection and other protective measures Public’s attention focused on infectious diseases Introduction of novel biomarkers (i.e., procalcitonin) Overall decreased antimicrobial consumption due to fewer patient consultations Reduction of critically ill patient transfer between countries
Increased rate of telemedicine in primary care (may favour both)	

## Data Availability

No new data were created or analyzed in this study. Data sharing is not applicable to this article.
